# Supplementary figures and images for: Mechanisms of maternal antibody interference with rotavirus vaccination
Source: EMBO J. 2025 Oct 14;44(22):6343–67. doi: 10.1038/s44318-025-00582-2 (PMC12623505; doi:10.1038/s44318-025-00582-2)

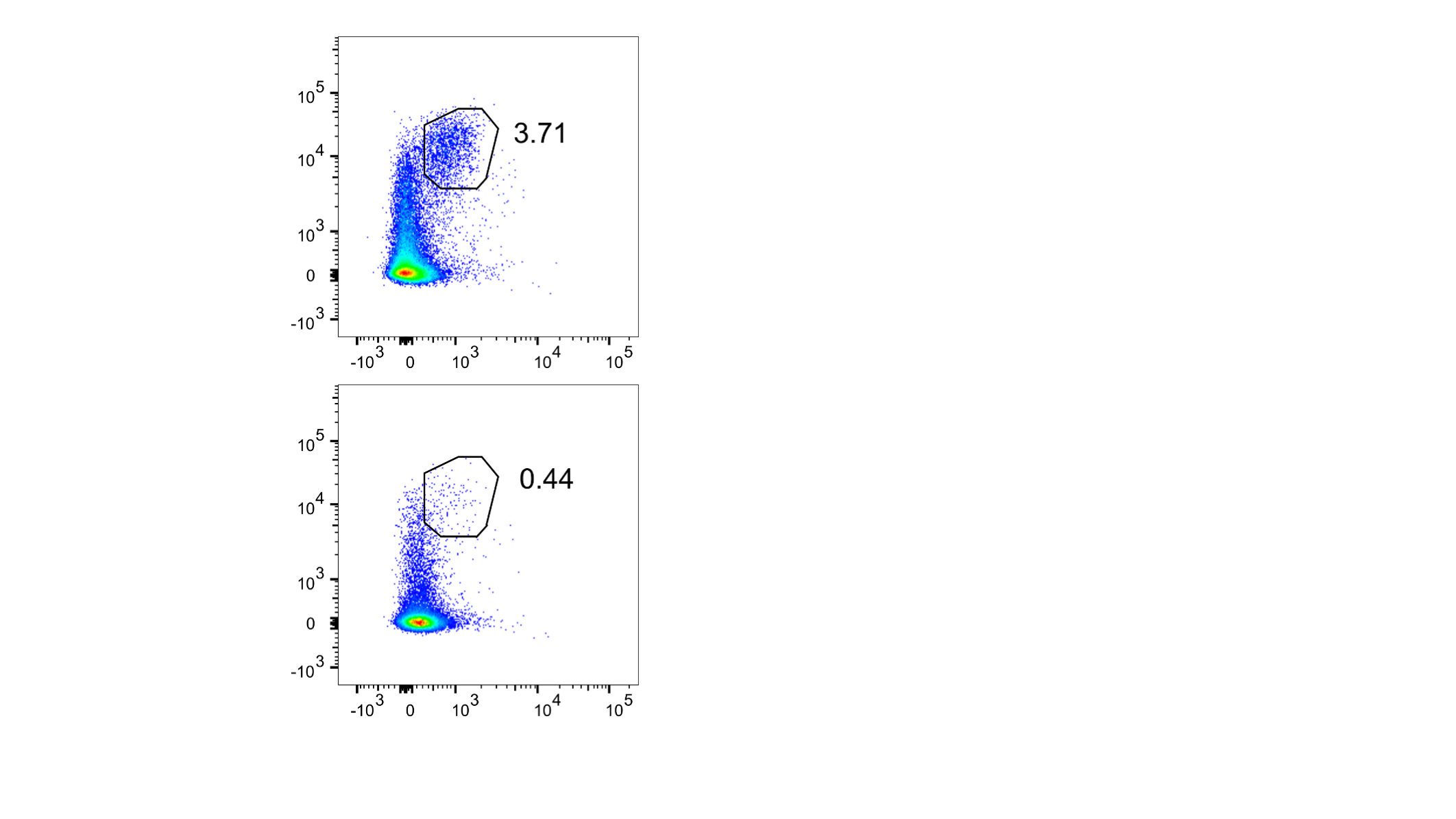

Supplement: Supplementary file 3 — Source data Fig. 2 [file 44318_2025_582_MOESM3_ESM.zip › Figure 2/2A/flow cytometry GC in MLN.tiff]

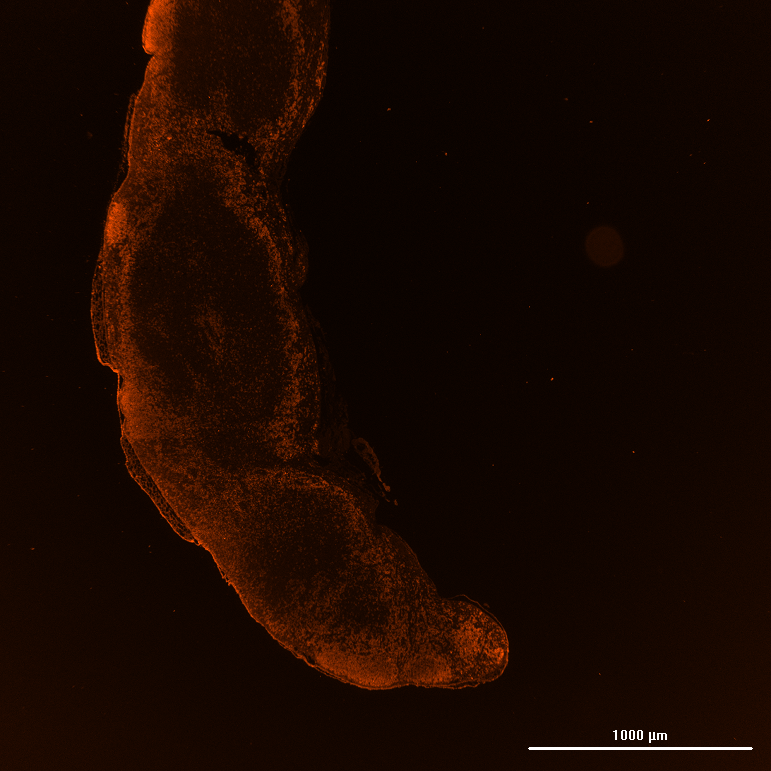

Supplement: Supplementary file 3 — Source data Fig. 2 [file 44318_2025_582_MOESM3_ESM.zip › Figure 2/2C/immunofluorescence TRITC B220 MLN GC MatAbs.png]

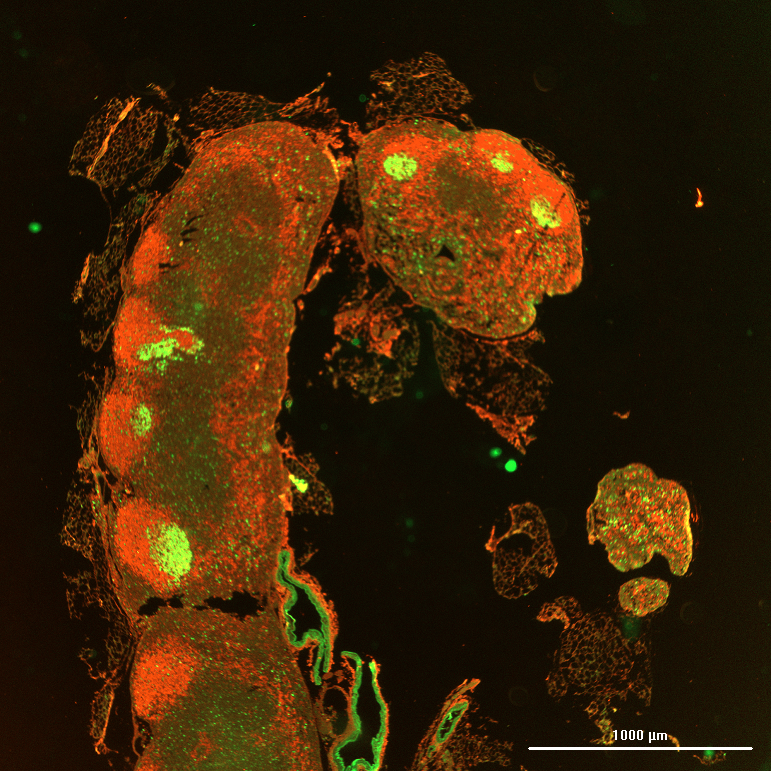

Supplement: Supplementary file 3 — Source data Fig. 2 [file 44318_2025_582_MOESM3_ESM.zip › Figure 2/2C/immunofluorescence GFP-TRITC merge MLN GC vaccine only.tiff]

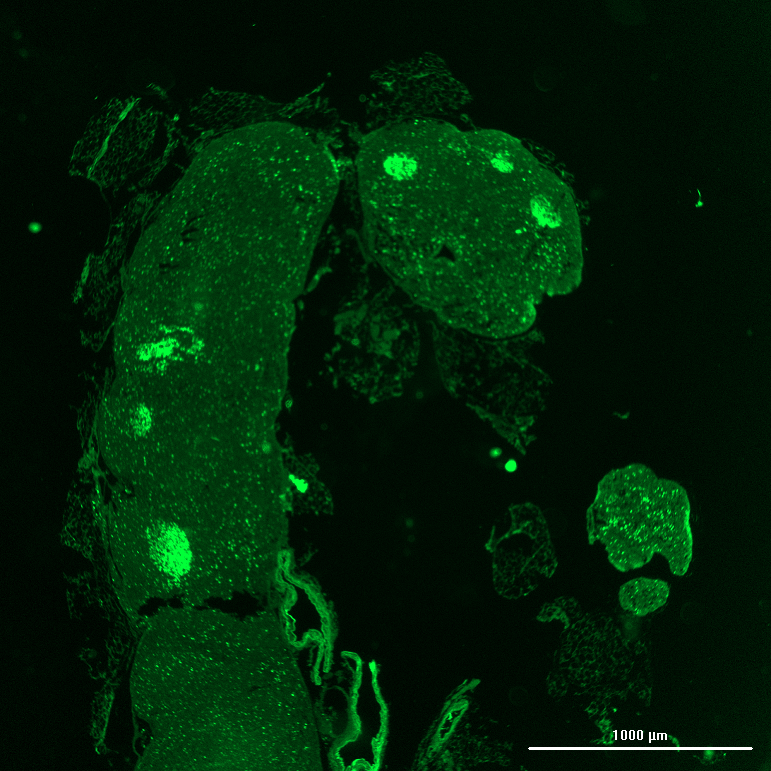

Supplement: Supplementary file 3 — Source data Fig. 2 [file 44318_2025_582_MOESM3_ESM.zip › Figure 2/2C/immunofluorescence GFP Ki67 MLN GC vaccine only A.png]

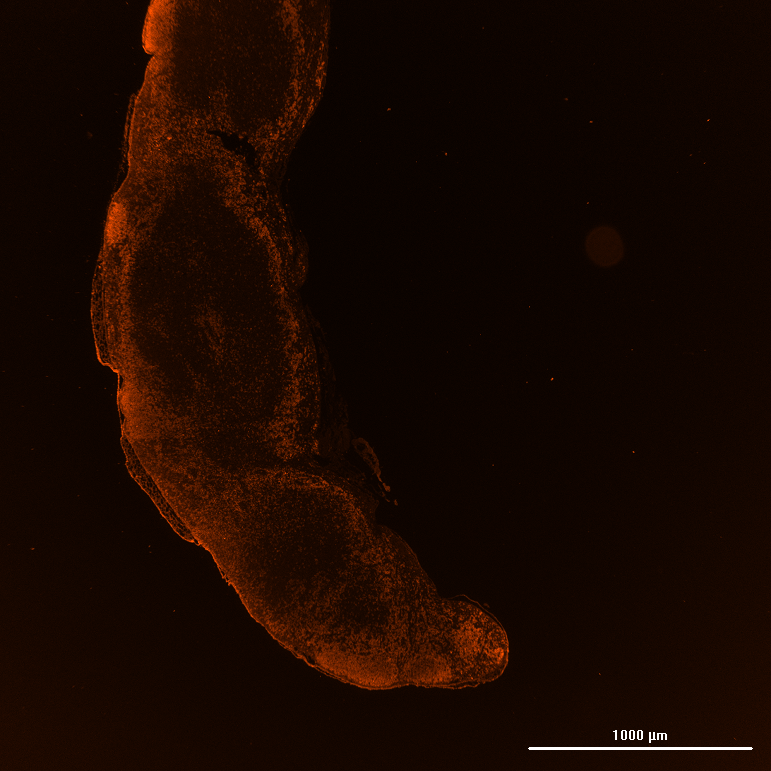

Supplement: Supplementary file 3 — Source data Fig. 2 [file 44318_2025_582_MOESM3_ESM.zip › Figure 2/2C/immunofluorescence TRITC B220 MLN GC MatAbs.tiff]

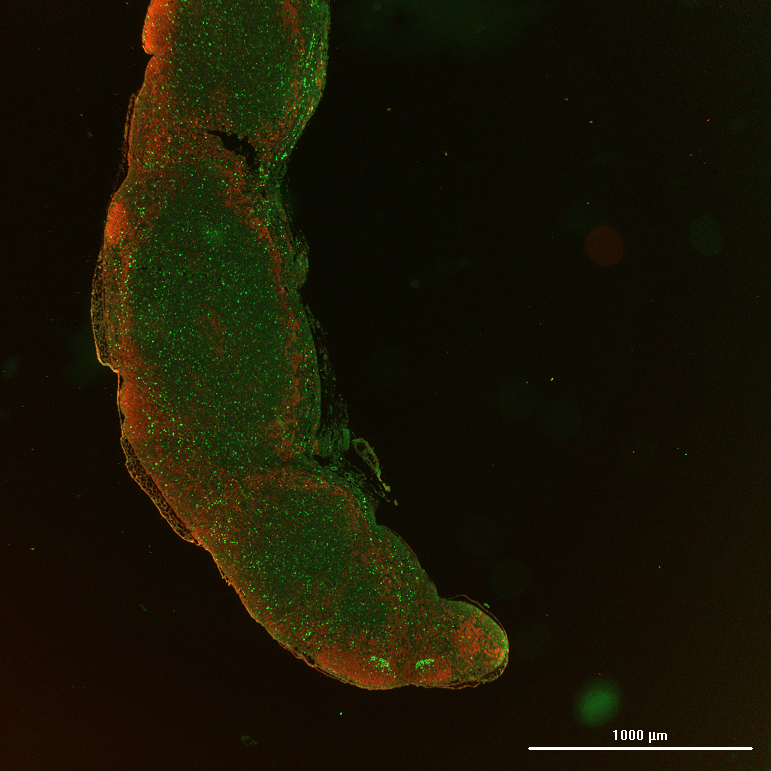

Supplement: Supplementary file 3 — Source data Fig. 2 [file 44318_2025_582_MOESM3_ESM.zip › Figure 2/2C/immunofluorescence GFP-TRITC merge MLN GC MatAbs.png]

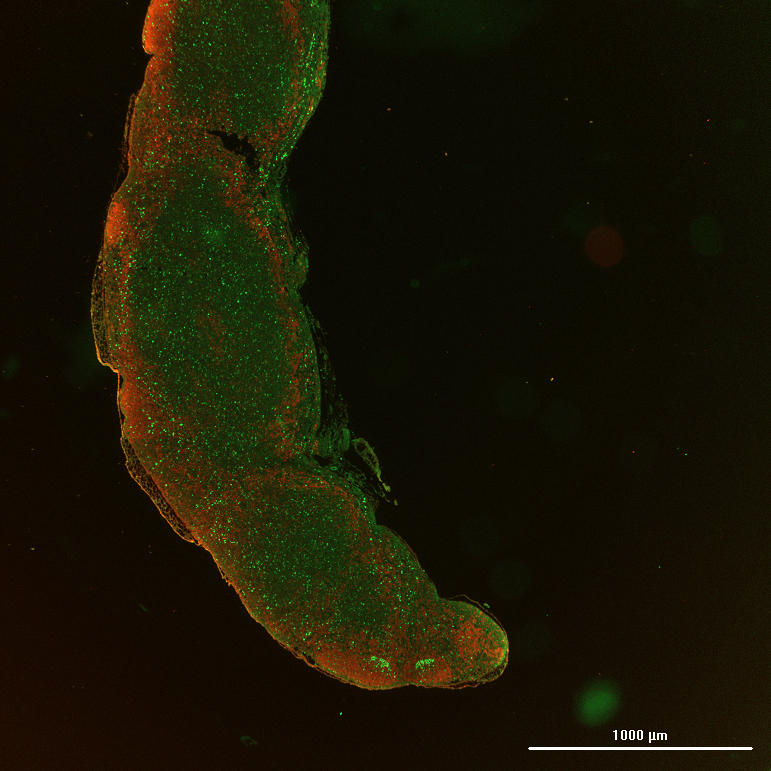

Supplement: Supplementary file 3 — Source data Fig. 2 [file 44318_2025_582_MOESM3_ESM.zip › Figure 2/2C/immunofluorescence GFP-TRITC merge MLN GC MatAbs.tiff]

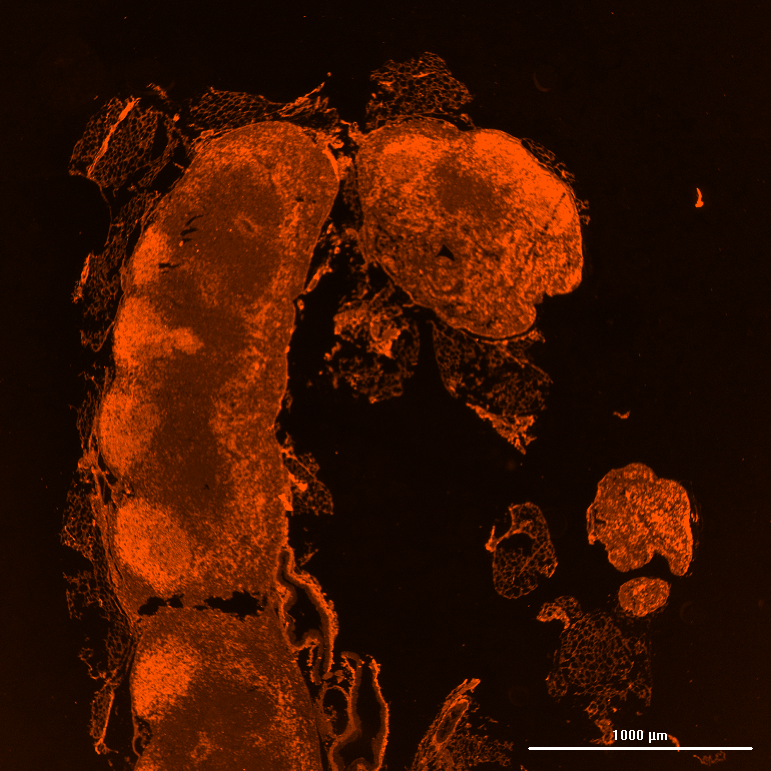

Supplement: Supplementary file 3 — Source data Fig. 2 [file 44318_2025_582_MOESM3_ESM.zip › Figure 2/2C/immunofluorescence TRITC B220 MLN GC vaccine only.png]

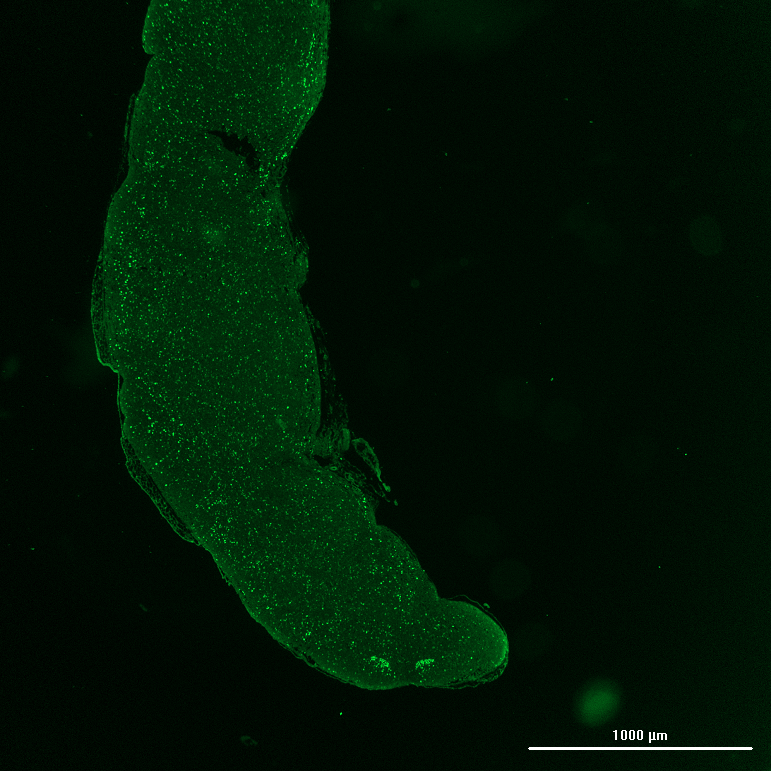

Supplement: Supplementary file 3 — Source data Fig. 2 [file 44318_2025_582_MOESM3_ESM.zip › Figure 2/2C/immunofluorescence GFP Ki67 MLN GC MatAbs.png]

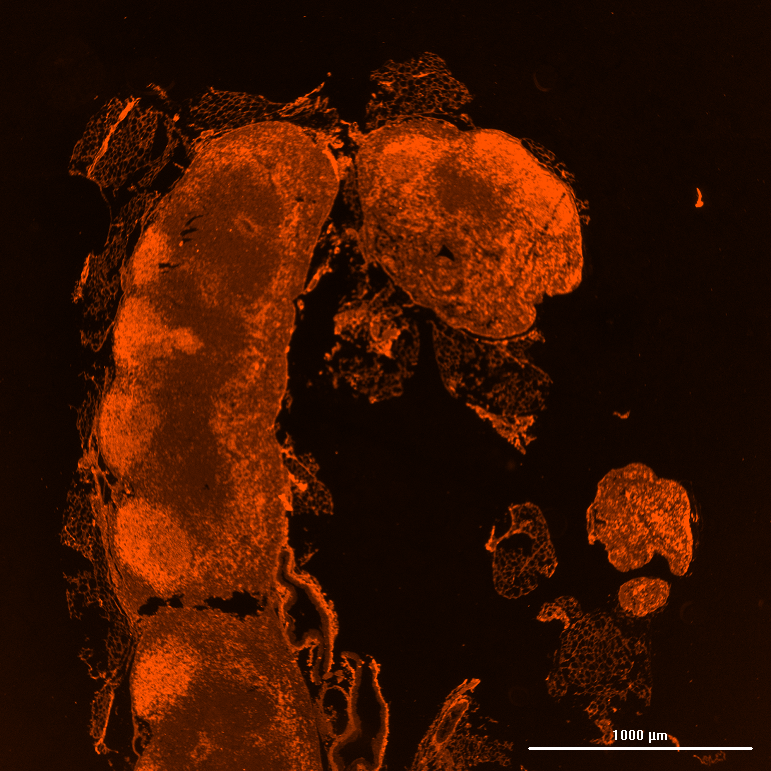

Supplement: Supplementary file 3 — Source data Fig. 2 [file 44318_2025_582_MOESM3_ESM.zip › Figure 2/2C/immunofluorescence TRITC B220 MLN GC vaccine only.tiff]

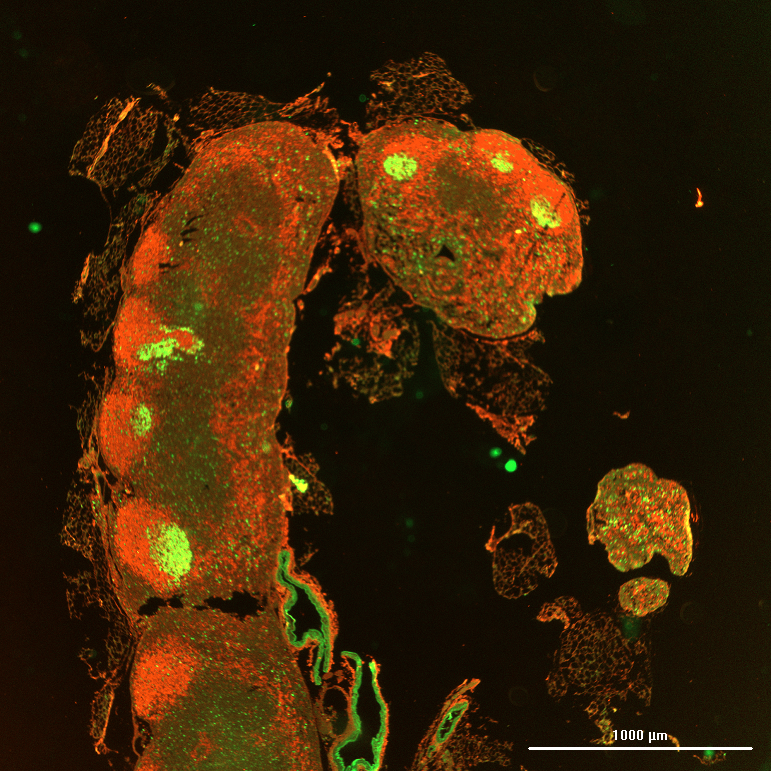

Supplement: Supplementary file 3 — Source data Fig. 2 [file 44318_2025_582_MOESM3_ESM.zip › Figure 2/2C/immunofluorescence GFP-TRITC merge MLN GC vaccine only.png]

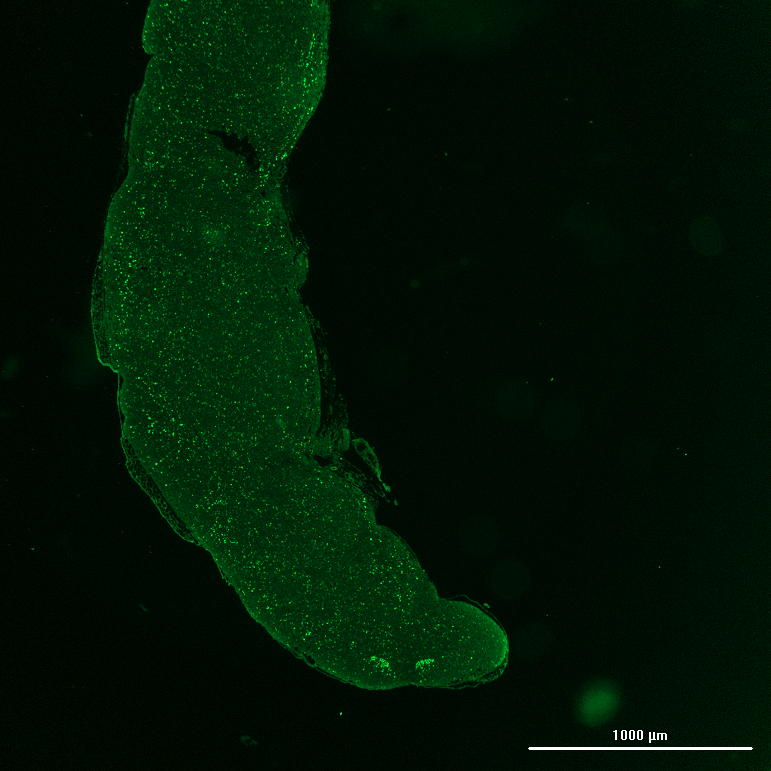

Supplement: Supplementary file 3 — Source data Fig. 2 [file 44318_2025_582_MOESM3_ESM.zip › Figure 2/2C/immunofluorescence GFP Ki67 MLN GC MatAbs.tiff]

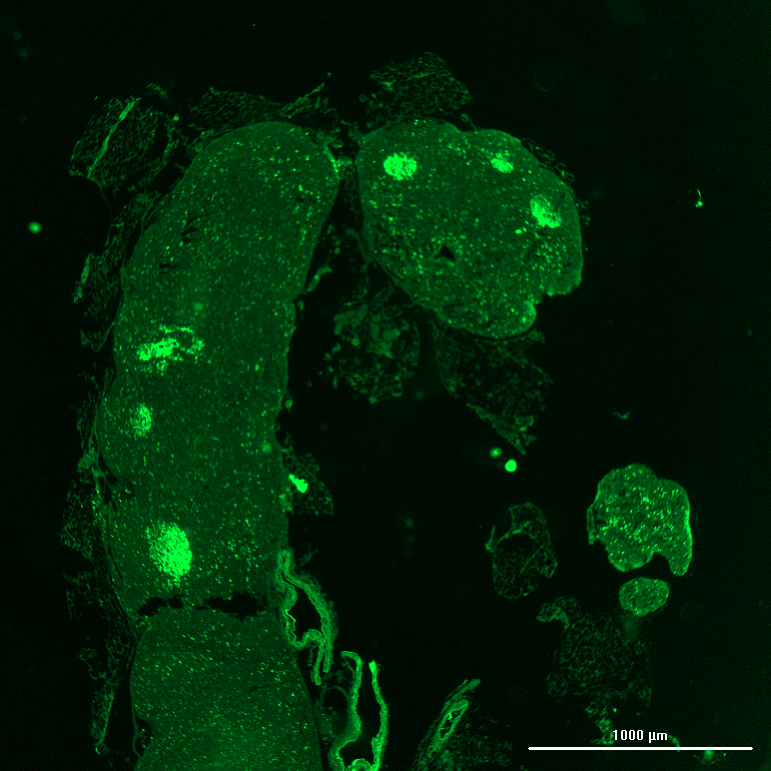

Supplement: Supplementary file 3 — Source data Fig. 2 [file 44318_2025_582_MOESM3_ESM.zip › Figure 2/2C/immunofluorescence GFP Ki67 MLN GC vaccine only.tiff]

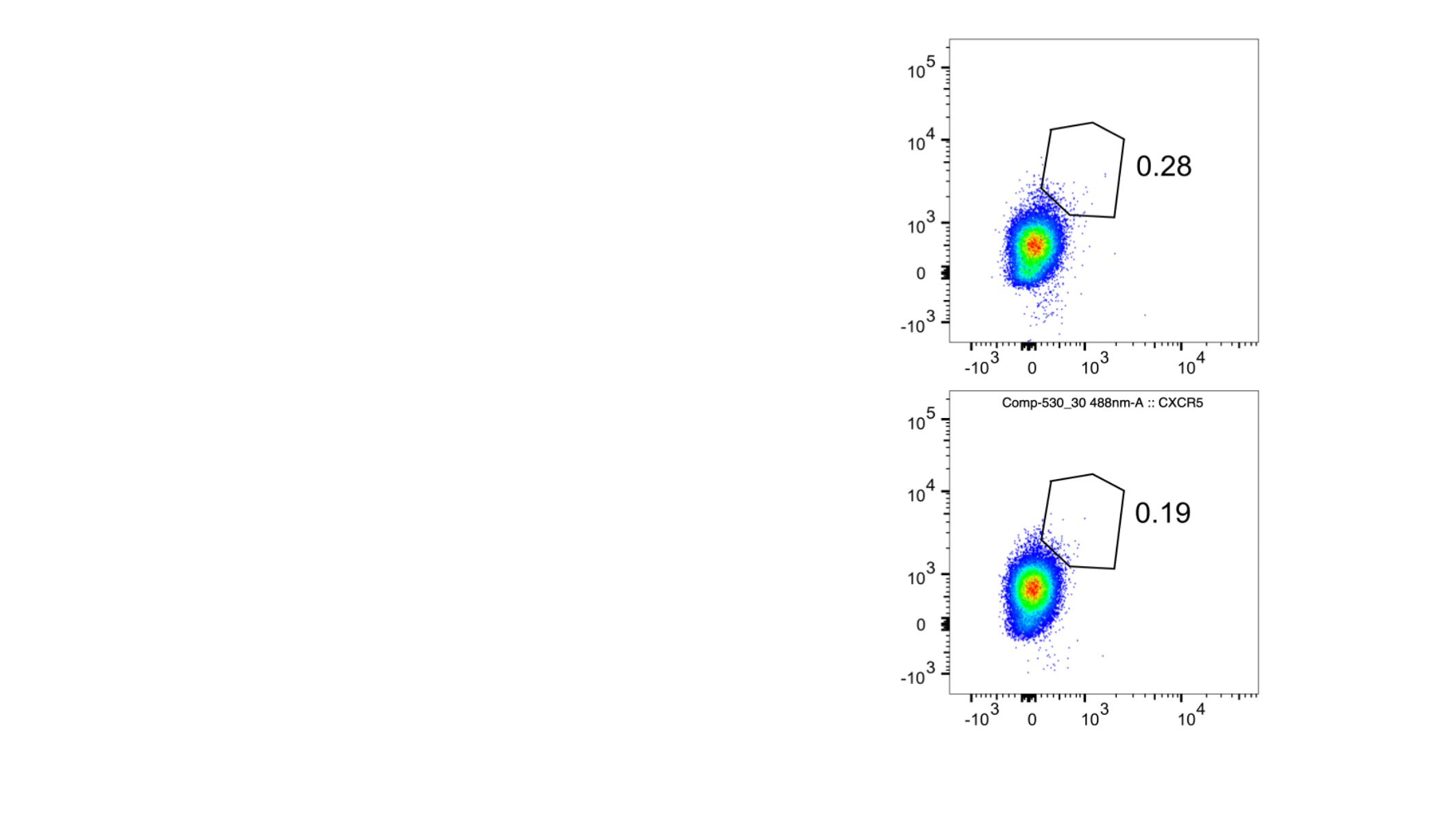

Supplement: Supplementary file 3 — Source data Fig. 2 [file 44318_2025_582_MOESM3_ESM.zip › Figure 2/2D/flow cytometry TFH in MLN.tiff]

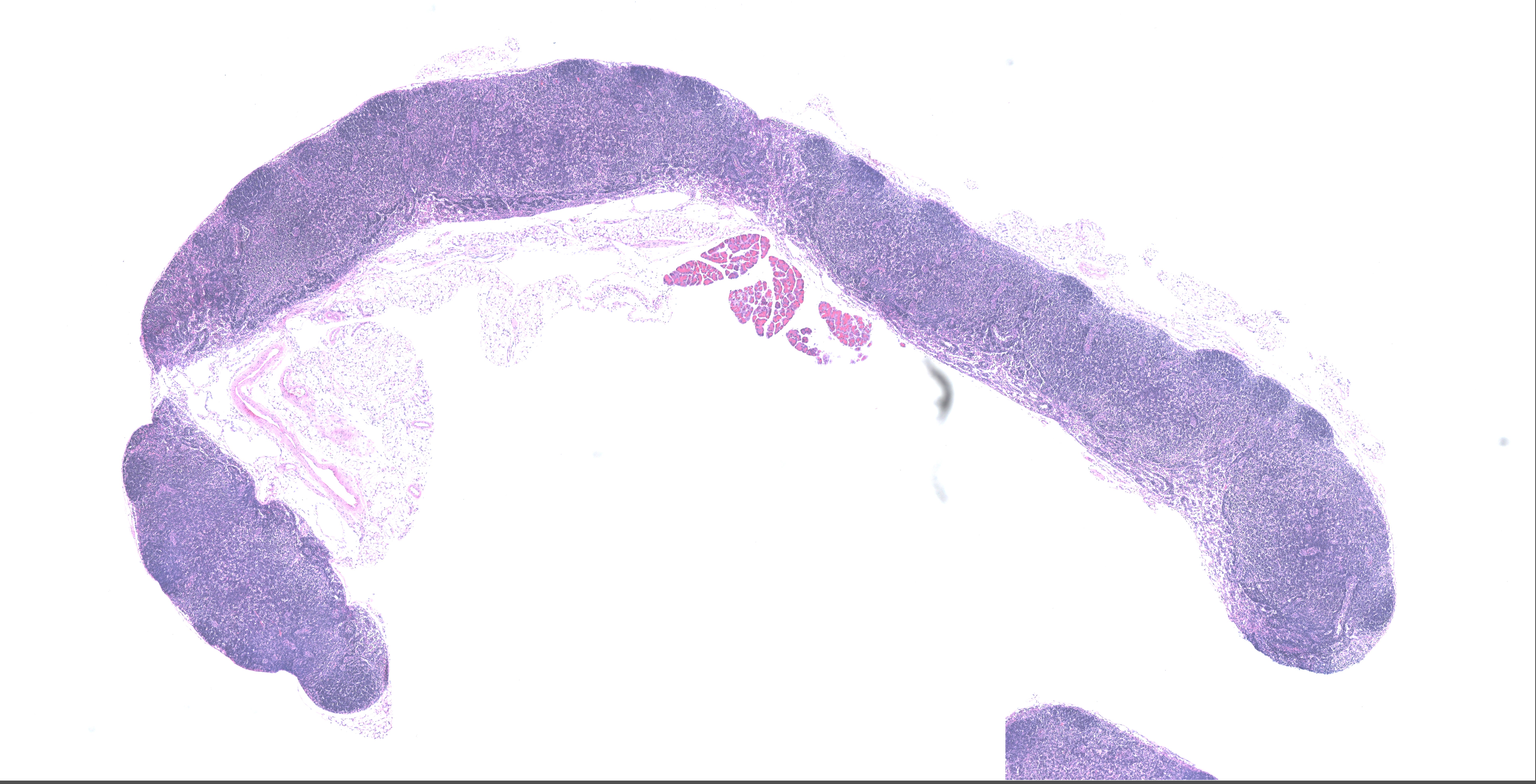

Supplement: Supplementary file 3 — Source data Fig. 2 [file 44318_2025_582_MOESM3_ESM.zip › Figure 2/2B/HandE staining MLN MatAb vaccine.jpg]

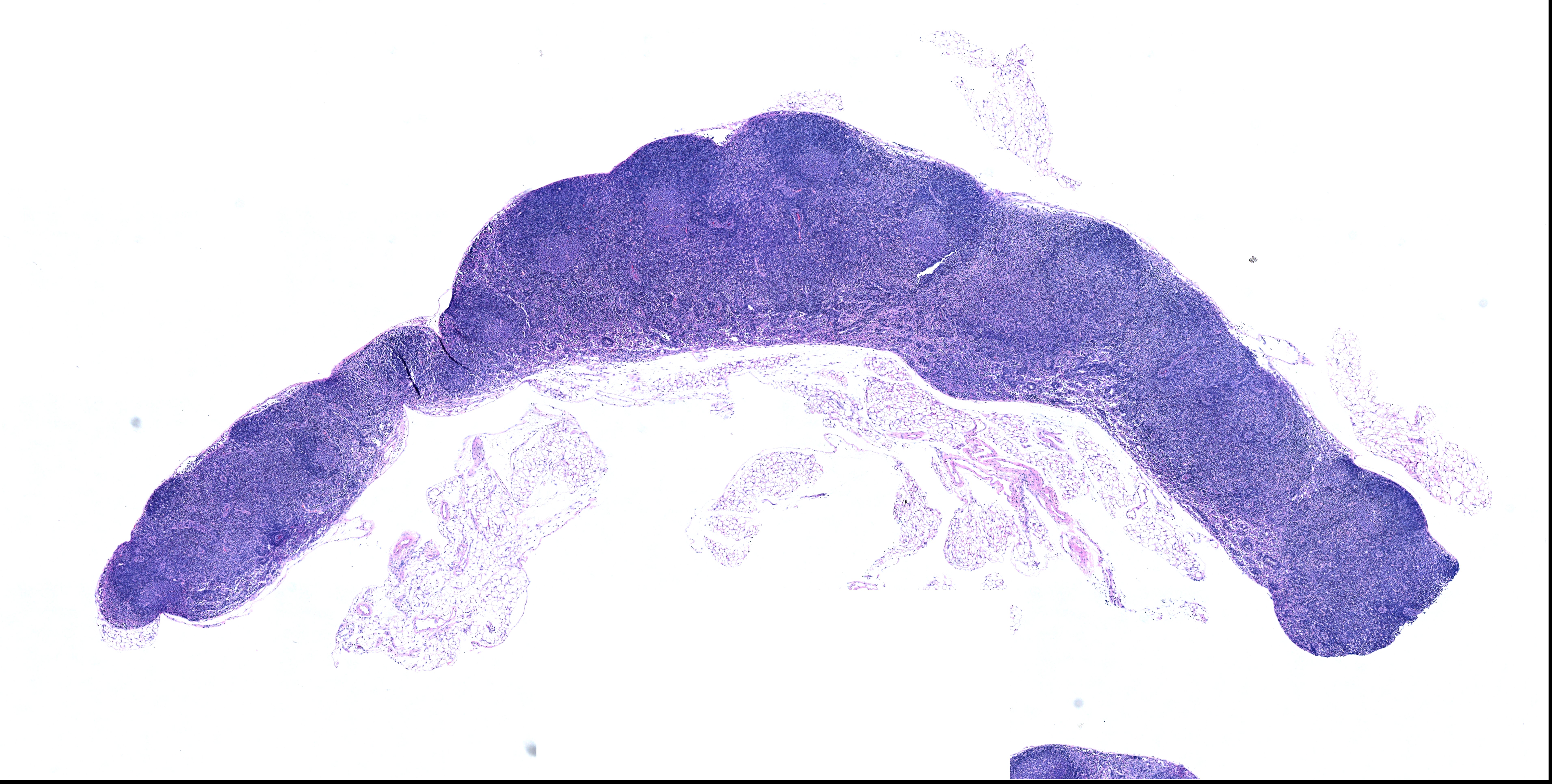

Supplement: Supplementary file 3 — Source data Fig. 2 [file 44318_2025_582_MOESM3_ESM.zip › Figure 2/2B/HandE staining MLN vaccine only.jpg]
