# Supplementary material for: Mechanisms of maternal antibody interference with rotavirus vaccination
Source: EMBO J. 2025 Oct 14;44(22):6343–67. doi: 10.1038/s44318-025-00582-2 (PMC12623505; doi:10.1038/s44318-025-00582-2)
Supplement: Supplementary file 11 — Expanded View Figures [file 44318_2025_582_MOESM11_ESM.pdf]

Expanded View Figures

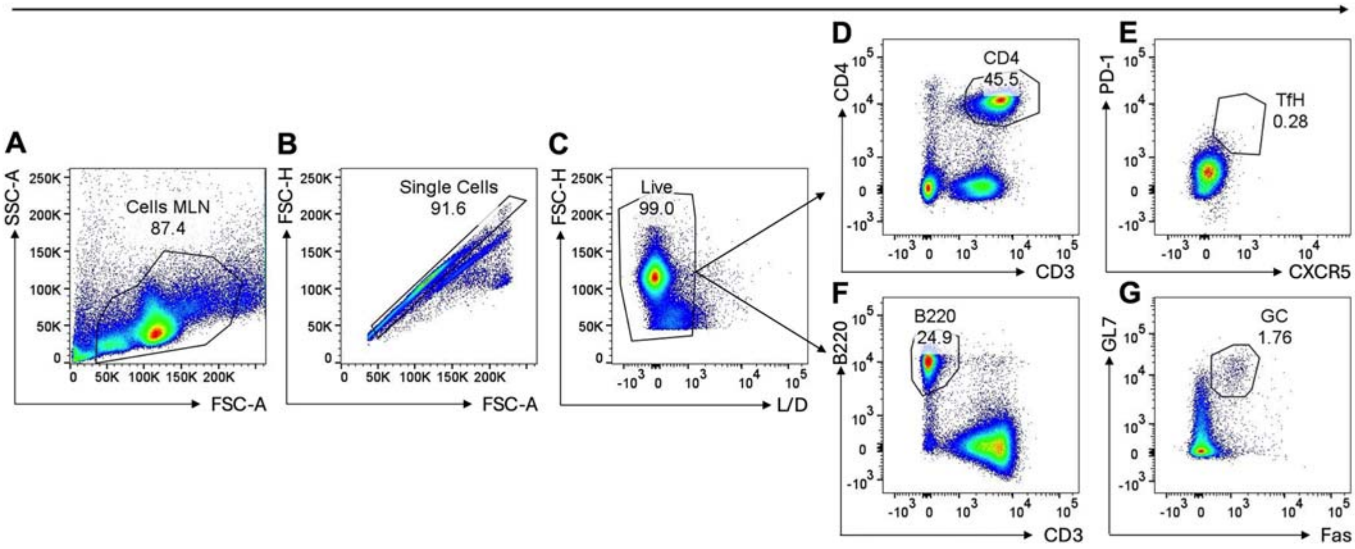

Figure EV1. Gating strategy for identification of Tfh cells and GC B cells by flow cytometry.

(**A**) Plot for forward versus side scatter, and mesenteric lymph node (MLN) cells gate. (**B**) Plot of forward scatter area as a function of forward scatter height within the MLN cells gate, and resulting single cells gate. (**C**) Viability dye (L/D) exclusion plot within the singlet gate, and resulting live cell gate. (**D**) Gating of CD4 cells, then gating of T follicular helper (Tfh) cells. (**E**) identified by PD-1 and CXCR5 within the CD4 gate. This panel is presented as a representative mouse in Fig. 2D. (**F**, **G**) Gating of B220+ B cells (**F**), then gating of germinal center (GC) B cells (**G**), identified by Fas and GL7 cells within the B220+ gate.

 © The Author(s)

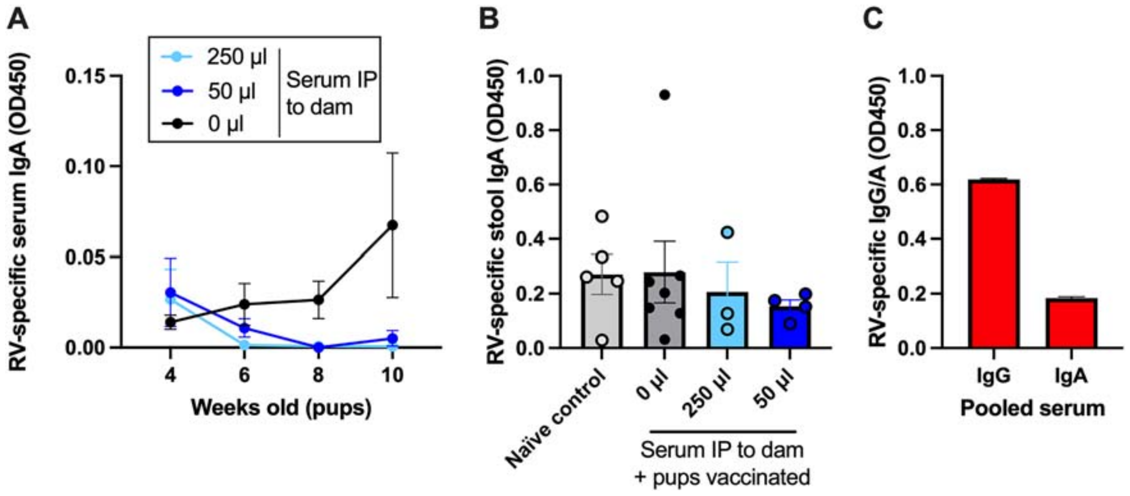

Figure EV2. Quantification of IgA in mouse stool and serum samples in the presence or absence of MatAbs.

(**A**) Longitudinal serum IgA titers following vaccination at 7 days old with different volumes of rotavirus-seropositive serum passively transferred to dams; $n = 3$, $n = 4$, and $n = 9$ for 250, 50, and 0 µL serum, respectively. (**B**) Stool IgA titers in adult mice from experiment (**A**) 14 days after challenge with rotavirus; $n = 5$, $n = 7$, $n = 4$, and $n = 3$ for naïve control, and 0, 50, and 250 µL, respectively. (**C**) Rotavirus-specific IgG and IgA ELISA of pooled serum used for passive transfer experiments, mean of $n = 2$ technical replicates shown. Data information: error bars indicate SEM. Source data are available online for this figure.

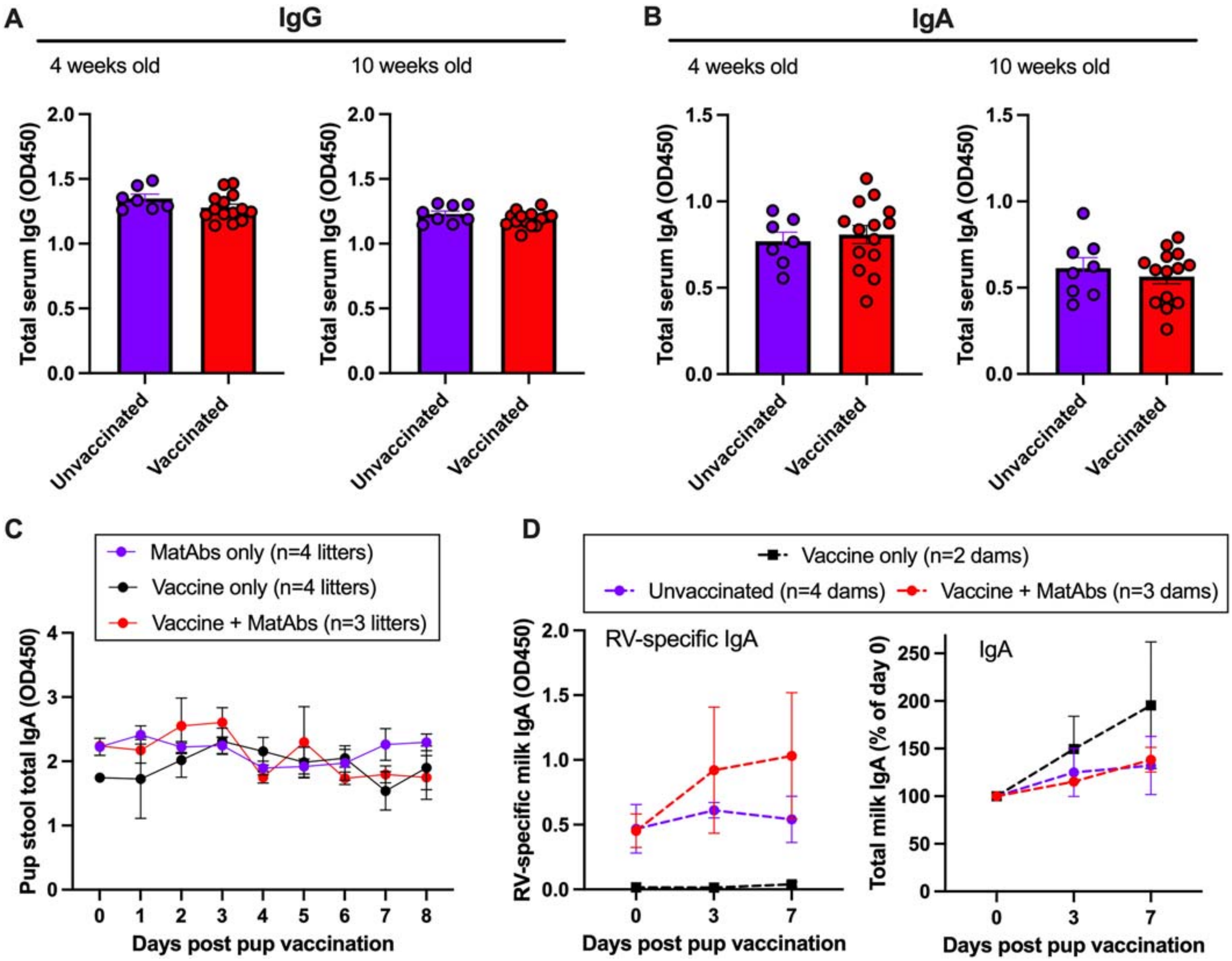

Figure EV3. Total IgG and IgA in serum, stool, and milk samples.

(**A**) Total IgG quantified by ELISA in serum samples from 4-week- and 10-week-old pups with MatAbs +/− rotavirus vaccination; $n = 7$ and $n = 14$ in unvaccinated and vaccinated pups at 4 wk and $n = 8$ and $n = 14$ in unvaccinated and vaccinated pups at 10 wk. (**B**) Total IgA quantified by ELISA in the same serum samples as in (**A**); $n = 7$ and $n = 14$ in unvaccinated and vaccinated pups at 4 wk and $n = 8$ and $n = 14$ in unvaccinated and vaccinated pups at 10 wk. (**C**) Total IgA ELISA in stool samples collected from pups 0 to 8 days after rotavirus vaccination. (**D**) Rotavirus-specific IgA and total IgA were quantified by ELISA in milk samples collected from dams at 0, 3, and 7 days after pup vaccination. Data information: error bars indicate SEM. Source data are available online for this figure.

 © The Author(s)

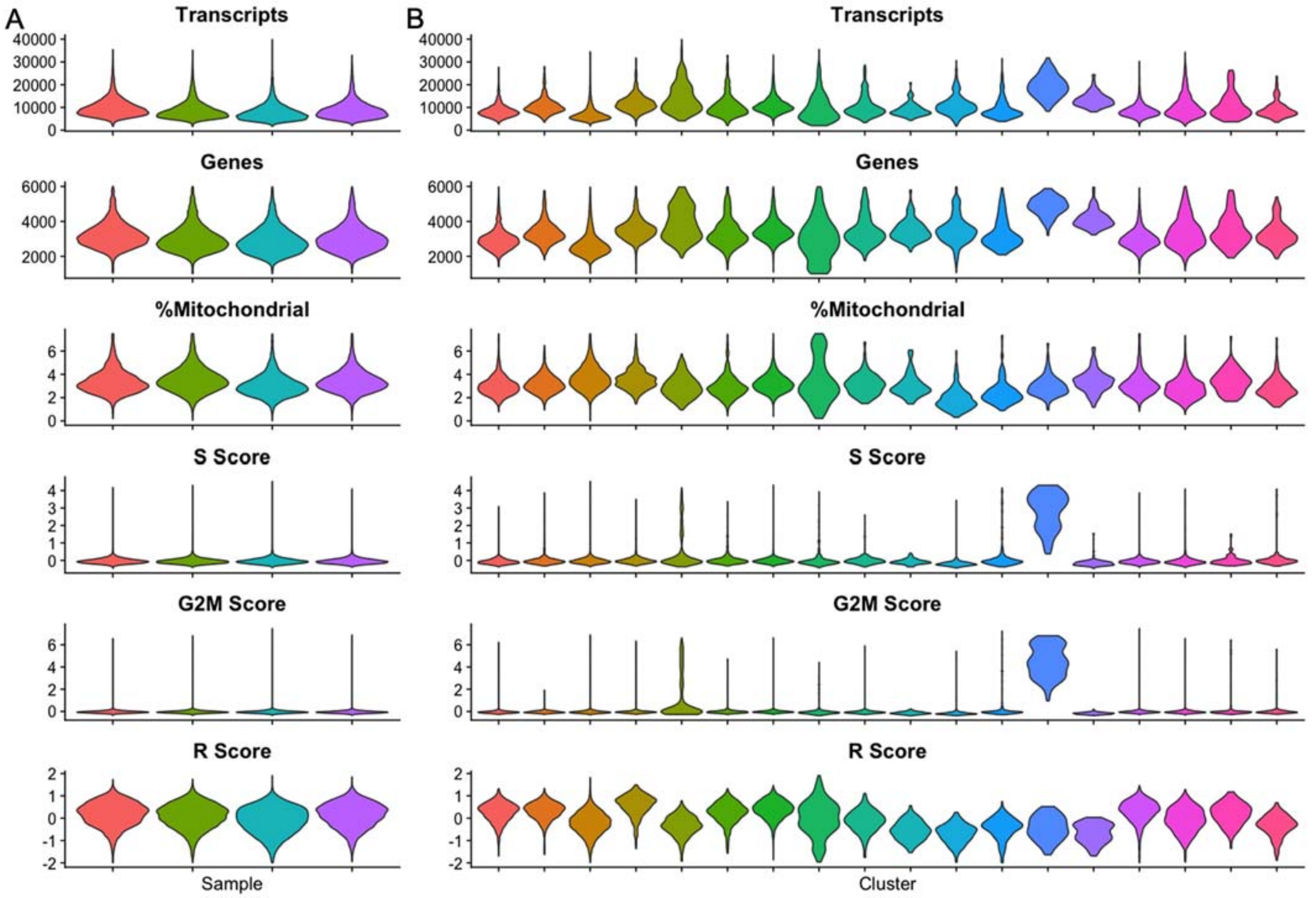

Figure EV4. Single-cell transcriptomics of the draining mesenteric lymph node.

(**A**) Violin plot showing quality control metrics (from top to bottom: transcripts per cell, genes per cell, percent mitochondrial reads per cell, S score, G2M score, and R score) for each sample ($n = 4$, x-axis) processed on the 10x Genomics Chromium instrument. (**B**) Violin plot showing quality control metrics for each cell type (x-axis) from MLN from pups vaccinated in the absence ($n = 2$) or presence ($n = 2$) of MatAbs.

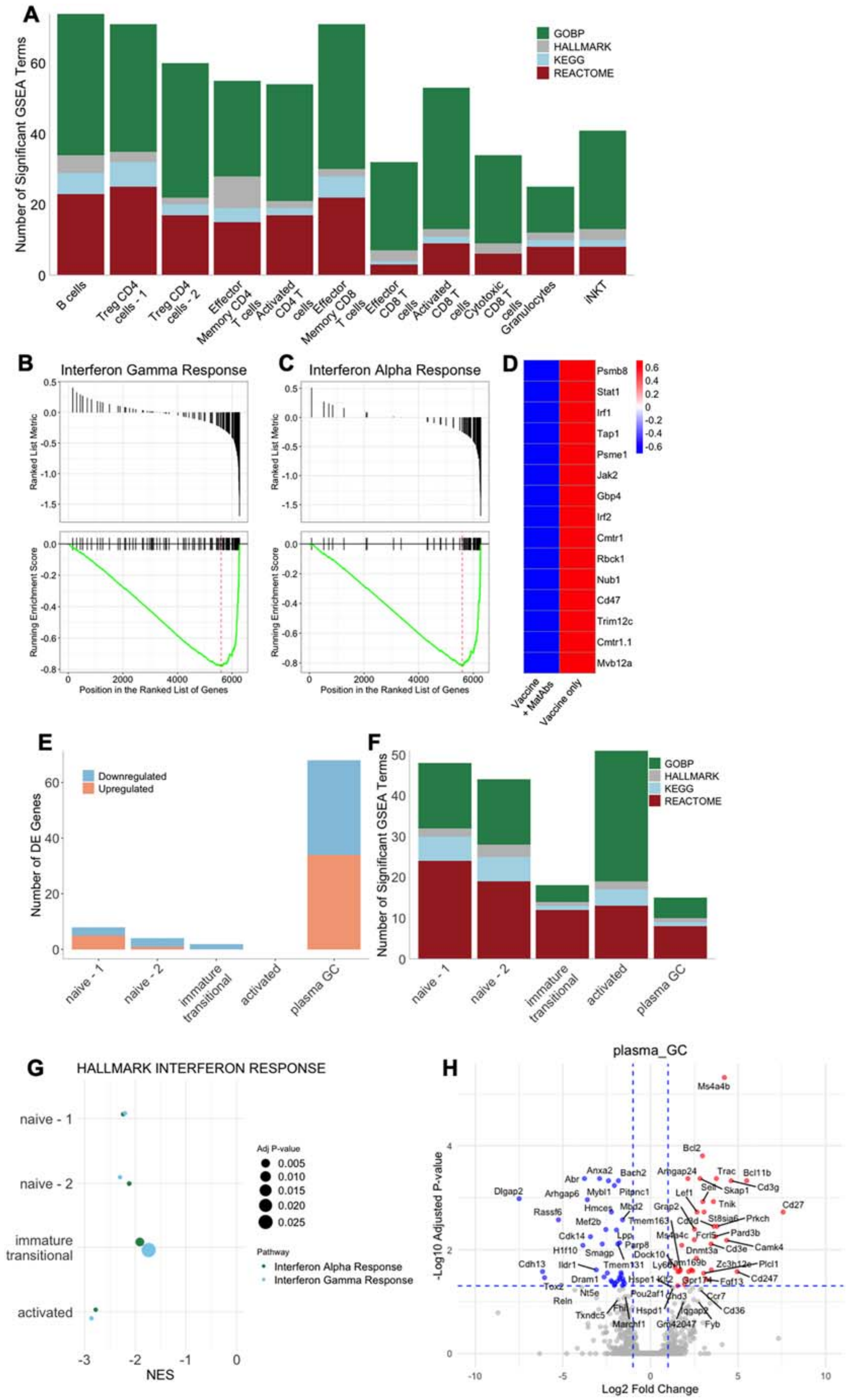
A
Number of Significant GSEA Terms
GOBP
HALLMARK
KEGG
REACTOME
B cells
Treg CD4 cells - 1
Treg CD4 cells - 2
Effector Memory CD4 T cells
Activated CD4 T cells
Effector Memory CD8 T cells
Effector CD8 T cells
Activated CD8 T cells
Cytotoxic CD8 T cells
Granulocytes
iNKT
B
Interferon Gamma Response
C
Interferon Alpha Response
Ranked List Metric
Running Enrichment Score
Position in the Ranked List of Genes
D
Psmb8
Stat1
Irf1
Tap1
Psme1
Jak2
Gbp4
Irf2
Cmtr1
Rbck1
Nub1
Cd47
Trim12c
Cmtr1.1
Mvb12a
Vaccine + MatAbs
Vaccine only
E
Number of DE Genes
Downregulated
Upregulated
naive - 1
naive - 2
immature transitional
activated
plasma GC
F
Number of Significant GSEA Terms
G
HALLMARK INTERFERON RESPONSE
Adj P-value
Pathway
Interferon Alpha Response
Interferon Gamma Response
NES
H
plasma_GC
-Log10 Adjusted P-value
Log2 Fold Change

Figure EV5. Differential gene expression in the draining mesenteric lymph node.

(**A**) Counts of the strongest significantly enriched gene sets with an absolute normalized enrichment score (NES) >2 across the largest cell clusters. (**B**) GSEA plot of hallmark interferon gamma response in activated CD4 T cells. (**C**) GSEA plot of hallmark interferon alpha response in activated CD4 T cells. (**D**) Heatmap of top ten core enrichment genes taken from the gene set Hallmark Interferon Gamma and Hallmark Interferon Alpha Response between vaccine + MatAb and vaccine only; expression values row-normalized; unique genes from both pathways plotted. (**E**) Counts of differentially expressed (DE) genes (y-axis) per B cell cluster, comparing vaccine + MatAb to vaccine only with absolute log2 fold change >1 and adjusted *p*-value ≤0.05. (**F**) Counts of the strongest significantly enriched gene sets with an absolute normalized enrichment score (NES) >2 across B cell clusters. (**G**) GSEA results showing downregulation of hallmark interferon alpha response and hallmark interferon gamma response in B cell clusters. Dots are sized to denote significance; the x-axis indicates NES. (**H**) Volcano plot for differentially expressed genes in vaccine + MatAb ($n = 2$) compared to vaccine only ($n = 2$) (MatAb/naïve) in plasma and GC cells. Up (red) and downregulated (blue) genes with absolute log2 fold change >1 and adjusted *p* value >0.05 shown; −log10 adjusted *p*-value shown. Data information: The Wald (default in DESeq2, version 1.44.0) test was used to generate *p*-values before Benjamini–Hochberg correction for multiple testing to generate adjusted *p*-values (**H**).

© The Author(s)
